# The intricate relationship between capillary refill time and systemic hemodynamics in septic shock

**DOI:** 10.1186/s13613-025-01563-8

**Published:** 2025-09-25

**Authors:** Glenn Hernandez, Eduardo Kattan, Gustavo Ospina-Tascón, Sebastian Morales, Nicolás Orozco, Gustavo García-Gallardo, Macarena Amthauer, Jing-Chao Luo, Jan Bakker

**Affiliations:** 1 Departamento de Medicina Intensiva, Facultad de Medicina, Pontificia Universidad Católica de Chile, Santiago, Chile; 2 Department of Intensive Care Medicine, Fundacion Valle del Lili, Cali, Colombia; 3 Translational Research Laboratory in Critical Care Medicine (TransLab- CCM), Universidad Icesi, Cali, Colombia; 4 Department of Critical Care Medicine, Sichuan Provincial People’s Hospital, Sichuan Academy of Medical Sciences, University of Electronic Science and Technology of China, Chengdu, China; 5 Department of Critical Care Medicine, Liangshan Yi Autonomous Prefecture First People’s Hospital, Xichang, China; 6 Department of Intensive Care, Erasmus MC University Medical Center, Rotterdam, the Netherlands

**Keywords:** Capillary refill time, Septic shock, Resuscitation, Fluid challenge, Mean arterial pressure challenge, Macro-to-microcirculatory coupling

## Abstract

The emergence and validation of capillary refill time (CRT) as a resuscitation target together with its rapid kinetics of response to increases in systemic blood flow makes it the ideal variable to assess clinical reperfusion and the status of macro-to-microcirculatory coupling in septic shock. Moreover, previous studies have shown that resuscitation can be safely stopped after CRT normalization, thus decreasing the risk of over-resuscitation. From a physiological point of view, CRT is a complex variable integrating microvascular flow and reactivity. Additionally, it may be understood as a dynamic test that evaluates the preservation or disruption of normal responses of the microcirculation to maintain blood flow after transient ischemic challenges. The relationship between systemic hemodynamics and CRT is complex. Indeed, single time-point asssessments of CRT are not able to predict absolute cardiac output values and this is logical since they belong to different phsyiological categories. An abnormal CRT may be explained by insufficient macrohemodynamic resuscitation but also by several derangements at the microvascular level that may preclude CRT normalization, thus signaling a state of macro-to-microcirculatory uncoupling. CRT response to an acute fluid or mean arterial pressure challenge, may not only reveal the adequacy of systemic blood flow but also contribute to tailor interventions to personalize septic shock resuscitation. The lack of CRT response to these challenges discloses a more complex pathophysiological condition that is associated with higher mortality. Further research efforts should be focused on better understanding the factors associated with CRT non-response as a first step to develop a more phsyiologically-based resuscitation, that could eventually improve outcomes.

## Background

Septic shock is associated with a high mortality risk of up to 30–60% [1, 2]. Multiple pathogenic factors, such as decreased vascular tone, hypovolemia, myocardial depression, endothelial dysfunction, coagulopathy, and microvascular abnormalities, can lead to progressive tissue hypoperfusion in the context of severe systemic inflammation [3]. Hemodynamic resuscitation is the key intervention to restore tissue perfusion in a timely manner and is essentially based on the administration of fluids and vasoactive drugs to improve systemic blood flow and perfusion pressure [1]. Macrohemodynamic optimization with these interventions is guided by escalating hemodynamic monitoring strategies tailored to the clinical context, with the ultimate goal of enhancing microcirculatory flow [4]. Improvements in perfusion may be assessed through dynamic variables such as capillary refill time (CRT), which serves as a bedside signal of the reperfusion process [1, 2, 5–7]. Initially described in the 1940’s, CRT has gained traction as a valuable clinical sign in the critical care literature [8]. Moreover, the emergence and validation of CRT as a resuscitation target is supported by strong epidemiological data, physiological rationale, and a major randomized controlled trial—ANDROMEDA-SHOCK [9].

In shock states a compensatory neurohumoral response diverts blood flow from regions as the skin, liver and splanchnic vascular bed towards more fundamental organs [10]. At this stage, skin blood flow becomes directly dependent on systemic blood flow, perfusion pressure, and adrenergic tone, and is further modulated by the status of microvascular reactivity [6]. Importantly, the skin provides a convenient window into local microcirculatory flow, and a successful resuscitation is suggested by the transition from a cold clammy skin to a warm vasodilatory state—as captured by CRT assessment [6, 11–13]. Therefore, a positive CRT response following resuscitative interventions may be a signal of a successful restoration of microcirculatory flow, whereas a lack of improvement may suggest severe microvascular dysfunction [11, 12].

Following the landmark ANDROMEDA-SHOCK trial, subsequent research has primarily focused on expanding the epidemiological evidence supporting the prognostic value of CRT across diverse clinical scenarios [12, 14, 15]. More importantly, recent efforts have addressed additional aspects that are far more challenging. First, to elucidate the physiological determinants of CRT as a pivotal test for microvascular flow and reactivity [16, 17]. Second, to better characterize the relationship between systemic hemodynamics and CRT under static [18] and dynamic conditions, including its response kinetics to different hemodynamic challenges such as—fluid boluses and vasopressor tests— as a tool to explore the status of macro-to-microcirculatory coupling [19].

In this narrative review, we explore these domains in depth. Our aim is to provide a holistic view on the position of CRT as a multidimensional monitoring variable capable of interrogating not only macrohemodynamics, microcirculatory flow and reactivity, but also macro-to-microcirculatory coupling during septic shock resuscitation.

### Physiological determinants of capillary refill time

**Fig. 1:**
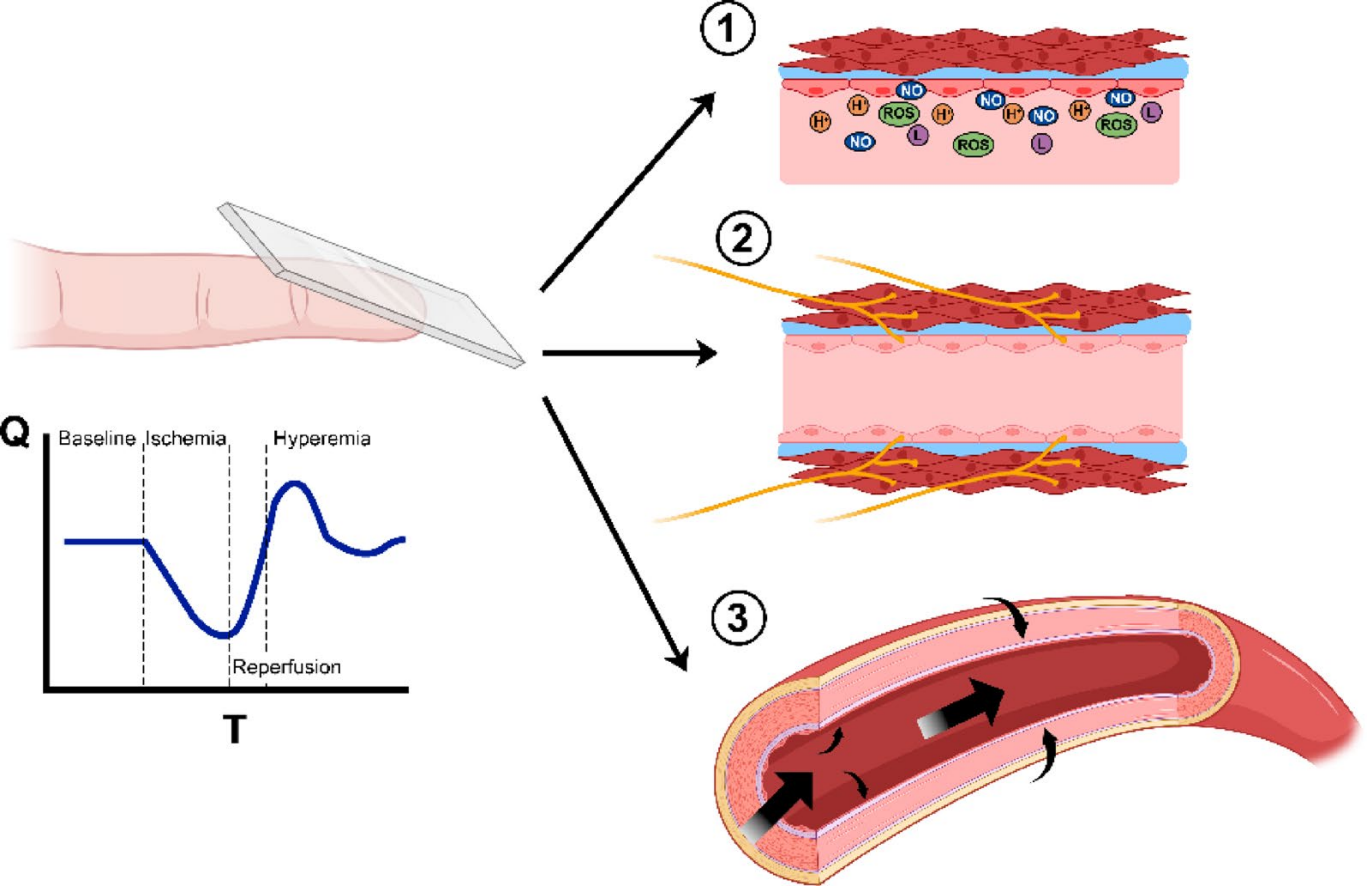
Potential determinants of capillary refill time as a test to assess microvascular reactivity. CRT could integrate three mechanisms involved in microvascular responses to transitory ischemia: (1) endothelial signals (mediated by nitric oxide, prostacyclin, reactive oxygen species, hydrogen ions, etc.) coming from cellular groups subjected to ischemia; (2) adrenergic sympathetic influx which regulates vascular tone in innervated vessels surrounded by smooth muscle; and (3) myogenic responses, which implies the constriction / relaxation of smooth muscle to adjust vessel diameter in response to intravascular pressure / flow variations. *Q* blood flow, *T* Time

Organ blood flow remains almost constant over a wide range of perfusion pressures as consequence of tightly regulated mechanisms in which diameter from first order to terminal arterioles respond to variations in transmural pressures, local and retrograde metabolic signals from parenchymal cells and endothelium, and vascular sympathetic influx. Figure 1 summarizes key determinants of CRT as a test of microvascular reactivity: endothelial signals (e.g., nitric oxide, prostacyclin) mediate vasodilation in response to hypoxia and shear stress; adrenergic influx reflects sympathetic vasoconstrictive tone, heightened during stress or hypoperfusion; and the myogenic response describes the intrinsic vascular smooth muscle reaction to pressure changes [20–22]. Remarkably, the autoregulatory blood flow response can interact with, and be overridden by, central neural-humoral mechanisms, but it is intrinsically independent of them.

The integrity of autoregulatory responses that preserve microvascular flow in response to changes in driving pressures and systemic flow can be assessed through any ischemia-reperfusion challenge. For example, this may be evaluated by measuring the recovery slope of tissue oxygen saturation following forearm or lower-extremity stagnant ischemia using near-infrared spectroscopy [23, 24] or by assessing microvascular flow by laser doppler flowmetry following a skin ischemic or thermal challenge [16]. In an analogous way, CRT may reflect the preservation or disruption of such mechanosensitive and autonomic mechanisms that regulate and try to maintain blood flow to the skin in response to variations in perfusion pressures and flow/shear stress following transient ischemia and subsequent vascular occlusion release. Further studies should asses if CRT can be used as a simple bedside test to evaluate microvascular reactivity following a brief ischemic challenge.

Nevertheless, other authors interpret CRT as a direct indicator of tissue perfusion, given its strong correlation with skin perfusion assessed by laser doppler flowmetry in critically ill patients [17], and its moderate association with blood flow of intra-abdominal organs during septic shock [25]. Interestingly, CRT decreases rapidly after passive leg raising [26] and fluid expansion [27] but rarely improves without simultaneous improvement in macrohemodynamics [28], which favors its close link with tissue perfusion. In addition, similar to other direct markers of altered microcirculation, CRT may remain prolonged despite significant increases in cardiac output (CO) and mean arterial pressure (MAP) [28].

On the other hand, CRT has traditionally been interpreted as an indicator of volume status and dehydration in pediatric populations [29]. However, its value as predictor of hypovolemia in adults [30, 31] and elderly humans [32] is highly debatable. In fact, during lower body negative pressure challenges that elicited significant reductions in arterial pressure in healthy volunteers, CRT paradoxically showed a significant shortening at maximum provocation [33]. This findings suggest that, rather than demonstrating hypovolemia, CRT changes could potentially reflect the preserved capacity of microvascular mechanosensitive mechanisms to adapt flow in response to an acute decrease of CO in hypotensive patients during pre-shock stages of hemorrhage [33].

In conclusion, CRT should be understood as a dynamic test that evaluates the preservation or disruption of normal responses of the microcirculation to maintain blood flow after transient ischemic challenges. Consequently, persistently abnormal CRT after initial fluid resuscitation may identify a subset of patients with more severe phenotypes of septic shock [34], potentially indicating the need for further intervention. Conversely, CRT normalization might indicate that resuscitation efforts can be safely discontinued [35], as a preserved microvascular reactivity likely guarantees the adequacy of tissue perfusion.

### CRT and systemic hemodynamics during septic shock resuscitation: a relationship conditioned by the status of macro-to-microcirculatory coupling

Since Shoemaker’s strategy to increase CO to supranormal values as a resuscitation endpoint was abandoned in the 1990’s, primary resuscitation goals have shifted towards the normalization of tissue perfusion variables by manipulating systemic blood flow [2]. This change has led to a concept of adequacy/inadequacy of CO to supply metabolic demands rather than a “normality” categorization. Consequently, the relationship between a downstream variable such as CRT with CO should be assessed under this same perspective. For instance, does a normal CRT reflect CO adequacy? Or conversely, does a prolonged CRT necessarily translate in an insufficient systemic blood flow? This shift in perspective has prompted researchers to explore the dynamic behavior of CRT during hemodynamic challenges, thereby further unraveling the complex mechanisms that govern macro-to-microcirculatory coupling [7].

Macro-to-microcirculatory coupling refers to the concurrent improvement in microcirculatory flow following macrohemodynamic resuscitation, (i.e., increased CO and/or MAP), thereby resolving tissue hypoperfusion [7, 36, 37]. In contrast, uncoupling describes the failure to achieve reperfusion despite macrohemodynamic resuscitation. In this case, further fluids or vasopressors may lead to over-resuscitation and associated morbidity [7]. One fundamental research question on macro-to microcirculatory coupling is which are the major determinants of uncoupling, since theoretically some of these determinants may be targeted by a better physiologically-oriented titration of fluids and vasopressors.

Since the pivotal study by De Backer et al. [38] that introduced hand-held intravital videomicroscopy to assess sublingual microcirculation in critically ill patients, numerous studies have described a range of abnormalities linked to endothelial inflammation [39] and demonstrated their prognostic relevance across different clinical contexts [40, 41], particularly in septic shock [42]. Several scoring systems have been proposed to quantify abnormalities in microvascular flow, perfused vessel density and heterogeneity [43]. Research has consistently shown that both severity and persistence of microcirculatory abnormalities are associated with mortality and organ dysfunction. These inflammation-related microcirculatory abnormalities were further linked by Ince et al. to the concept of “loss of hemodynamic coherence”, a phenomenon analogous to macro-to-microcirculatory coupling [37]. Moreover, Ince et al. described four types of abnormalities potentially involved on this uncoupling, some of which may eventually be iatrogenic in nature and related to fluid overload or venous congestion [37]. In other words, persistent microcirculatory derangements may underlie the failure to achieve adequate reperfusion despite apparent optimization of systemic blood flow.

**Fig. 2:**
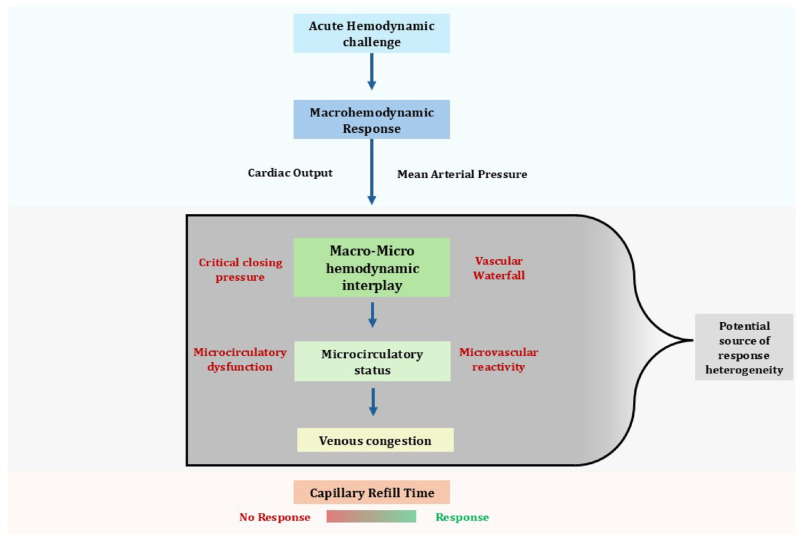
Potential mechanisms associated with macro-to microcirculatory uncoupling as linked to capillary refill time response

However, the limited availability of hand-held intravital videomicroscopy, combined with growing evidence supporting CRT as representing a more extensive microcirculatory territory like the skin, has opened new windows to explore the status of macro-to-microcirculatory coupling at the bedside. In line with this, a more dynamic view on the relation between systemic hemodynamics and the microcirculation has emerged as will be discussed below. Additionally, alternative explanations for uncoupling have been proposed. These include impaired microvascular reactivity resulting from progressive endothelial dysfunction [16], as well as an unbalance between macrohemodynamic and microhemodynamic variables—such as critical closing pressure, the arterial pressure below which blood flow stops despite a persistent pressure gradient between arteries and veins, and the vascular waterfall, a phenomenon in which vascular pressures drop abruptly from the critical closing pressure to the mean systemic filling pressure. In this context, once arterial pressure exceeds the critical closing pressure, blood flow becomes independent of downstream (venous) pressure, resembling flow over a waterfall edge [44–46]. Recent studies have introduced methods to assess these microhemodynamic variables, which although technically complex, may help explain the heterogenous response of the microcirculation to different levels of MAP [37, 38]. An expanded overview of potential mechanisms associated with macro-to microcirculatory uncoupling is shown in Fig. 2. Notably, the role of venous congestion has not been deeply explored but may be a treatable and easily recognized condition contributing to uncoupling.

### CRT and systemic hemodynamics during septic shock resuscitation: a holistic and dynamic view

Whether CRT provides meaningful insights into systemic hemodynamics depends on several key considerations. Fundamentally, systemic hemodynamics and CRT belong to two different physiological categories [47]. Concerning the first, in practical terms, CO, MAP, and their determinants can be readily monitored and manipulated through therapeutic interventions to improve global perfusion and microcirculatory flow during septic shock resuscitation. Nonetheless, targeting CO as a unique resuscitation endpoint has not demostrated beneficial clinical effects [48]. In contrast, CRT is a perfusion-related variable reflecting the status of an extensive microcirculatory territory, where ischemia/reperfusion processes can be assessed in real-time. Indeed, several observational studies have shown that CRT normalization after initial resuscitative interventions such as fluid loading is associated with a better prognosis as compared to patients with persistently abnormal peripheral perfusion [5, 12, 49]. Moreover, the ANDROMEDA-SHOCK trial found that patients who presented with a normal CRT at baseline, despite meeting septic shock criteria, exhibited a significantly lower 28-day mortality (27% vs. 43%) compared to patients with abnormal CRT [34]. Furthermore, among patients randomized to the CRT-guided resuscitation arm, those who achieved CRT normalization within two hours were able to safely stop resuscitation. In contrast, patients in the lactate-guided group were required to continue resuscitation if lactate remained elevated, even when CRT was normal—resulting in a higher overall mortality [35].

These and other findings suggest that a normal CRT during septic shock resuscitation reflects that systemic blood flow is adequate, and that active resuscitation may be safely stopped. Another interesting dimension is to introduce a dynamic view based on the rapid kinetics of response of CRT to common resuscitative interventions such as fluid or MAP challenges.

### The effect of a fluid challenge on CRT

Given that a fluid bolus is a primary intervention to enhance CO and improve tissue perfusion, several recent studies have examined how CRT evolves in response to a fluid challenge (Table 1). Jacquet-Lagrèze et al. showed that a reduction in CRT observed during a passive leg raising (PLR) test was predictive of CRT improvement after a subsequent fluid challenge in 15 of 34 cases [26]. In a separate study, Raia et al. administered a 500 mL fluid bolus to 29 patients and monitored CRT every two minutes post-infusion. Responders —defined as those with a CRT reduction over 0.2 s— accounted for 23 patients, in whom CRT significantly improved within 6–8 min and remained stable at 30 min [27]. Similarly, Fage et al. explored the effects of a 500 ml fluid bolus in 33 patients, finding a meaningful CRT reduction (over 23%) only in those with prolonged baseline CRT and an associated increase in CO [28]. However, CRT improved in some patients without a CO response and failed in others despite a rise in CO, thus CO was not an independent predictor of CRT change, questioning a direct relationship. In a more recent study, our group also evaluated the impact of a 500 ml fluid challenge in nine patients exhibiting abnormal CRT at baseline. A positive response—defined as a CRT reduction of more than one second—was observed in seven patients, though this was not consistently associated with a fluid challenge-induced change in CO [18].

**Table 1:** Effect of fluid and mean arterial pressure challenges on capillary refill time and macrohemodynamic variables

Study	N° of patients	Baseline CRT (s)	Baseline CO/CI (CO: l/min or CI: l/min/m^2^)	Fluid bolus characteristics	Post challenge CRT (s)	CRT responders (%)	CO/CI post fluid bolus (CO: l/min or CI: l/min/m^2^)
Fluid Challenge							
Raia et al. 2022 [20]	29	3.8[2.6–4.1]	CI: 2.8 [2.2–3.7]	500 ml of crystalloid in 15 min	2.5 [1.4–3.4]	23/29	CI: 3.4 [2.3–4.3]
Hernandez et al. 2024 [10]	9	5.0 [3.5–7.6]	CO: 5.7 +- 1.7	500 mL of crystalloid in 30 min	4 [2.4–5.1]	7/9	CO: 6.3 +-2.0
Jacquet Lagreze et al. 2019 – Responders [19]	15	3.6 [2.8-6.0]	CI: 2.1 [1.8–2.9]	500 ml of any fluid	2.5 [2.1–3.2]	15/15	CI: 3.0 [2.7–3.2]
Jacquet Lagreze et al. 2019– Non responders [19]	19	2.6 [2.3–3.3]	CI: 2.4 [2.1–3.3]	500 ml of any fluid	2.6 [2.0-3.1]	0/19	CI: 3.1 [2.6–3.4]
Fage et al. 2023 [21]	33	5.2 [3.2–7.4]	CI: 2.6 [2.1-3.0]	500 mL of crystalloid in 15 min	4.2 [2.8–5.9]	10/33	CI: 2.91[2.3–3.5]

Study	N° of patients	Baseline CRT (s)	Baseline NE dose (mcg/kg/min)	Drug titration characteristics	Post challenge CRT (s)	% CRT responders	CO effect
Mean arterial pressure challenge							
Fage et al. 2023 [21]	36	2.7 [1.6–4.9]	0.23 [0.08–0.51]	Norepinephrine was titrated by attending clinician. Post challenge, MAP achieved was of 85 [80–103] mmHg.	2.3 [1.3–4.1]	13.9% (5/36)	CI: 2.9 to 2.9 l/min/m^2^ (*p* < 0.0001)
Hernandez et al. 2024 [10]	25	4.0 [3.3–5.6]	0.18 + – 0.13	Norepinephrine was titrated until MAP 80–85 mHg was reached. Post challenge, MAP was of 84 [82–87] mmHg.	3 [2.6-5]	44%	CO: 4.7 to 5.0 l/min (*p* = 0.13)

*CRT* Capillary refill time, *CO* cardiac output, *CI* cardiac index, *MAP* mean arterial pressure

Data is presented as median [interquartile range] or mean +- standard deviation according to original publication

Taken together, these findings suggest that a fluid challenge can lead to rapid CRT improvement in most fluid-responsive patients. However, the presence of non-responders highlights the potential dissociation between macrohemodynamic and microcirculatory responses. The precise relationship between CO increases and CRT dynamics, particularly the durability of CRT improvement, remains unclear. In some cases, even a transient CO rise results in sustained CRT normalization, suggesting a possible microcirculatory recruitment. However, it is not well established whether a fluid bolus exerts direct microvascular effects —such as modulating endothelial function or local perfusion dynamics—that may influence CRT independently of global hemodynamic parameters [50]. In a recent proof-of-concept study, albumin infusion in patients with persistent peripheral hypoperfusion produced a higher rate of CRT normalization than saline and was accompanied by larger reductions in mottling score, indicating improved peripheral perfusion independently of systemic hemodynamic changes [51]. The authors speculated that a direct effect at the microvascular level could drive these results [51]. These results align with earlier clinical observations in septic shock, where albumin enhanced sublingual microvascular prefusion, including increased density of perfused capillaries and improved flow homogeneity, in comparison with crystalloids [42, 52]. These findings suggest that fluid therapy may alter CRT by targeting endothelial dysfunction rather than macrohemodynamics alone, but further research is needed.

### The effect of a MAP challenge on CRT

MAP serves as the primary driving force behind microvascular perfusion and is essential for maintaining adequate tissue oxygen delivery [12, 46]. Current guidelines recommend maintaining MAP above 65 mmHg in patients with septic shock [1, 53]. However, there is ongoing debate regarding whether higher MAP targets could further optimize tissue perfusion during shock. Several observational and interventional studies investigating elevated MAP thresholds have yielded inconclusive or conflicting results regarding their effect on key clinical outcomes [54–58]. Furthermore, targeting MAP alone may not adequately address the complex physiology of tissue perfusion in shock states, as aforementioned. As such, recent epidemiological analyses have suggested that lower MAP targets may be associated with reduced mortality in vasodilatory shock, although the causality of this relationship remains uncertain [59].

From a physiological standpoint, the impact of increasing MAP on microcirculatory flow is not uniform and depends on the interaction between systemic perfusion pressure and the effect on microvascular driving pressure. Research by Thooft et al. [60] and Dubin et al. [61] demonstrated that raising MAP using norepinephrine in septic patients resulted in highly variable changes in sublingual microvascular flow. Indeed patients with impaired microvascular flow at baseline tended to improve it, while others with preserved microvascular flow worsened this variable at higher MAP levels. In line with these findings, our recent study confirmed that elevating MAP to the same target value elicited heterogeneous effects on CRT —with some patients improving and others deterioraring CRT- after this MAP challenge (Table 1) [18].

The reason why some septic shock patients experience improvements in microcirculatory flow or CRT after a MAP challenge, while others show deterioration, is not fully understood. One potential explanation is that it may be related to the status of microvascular reactivity, where patients with more severe endothelial dysfunction are not able to improve CRT irrespective of macrohemodynamic optimization. Another hypothesis relates to an imbalance between the rise in MAP achieved through vasopressors and its simultaneous effect on downstream microhemodynamic variables—such as the critical closing pressure —subsequently affecting tissue perfusion pressure or the waterfall, as suggested by a recent study of Andrei et al. in vasoplegic patients after cardiac surgery [62]. Ongoing research is expected to provide further insight on this issue, particularly through the development of less complex and minimally invasive methods for assessing these microhemodynamic variables at the bedside.

**Fig. 3:**
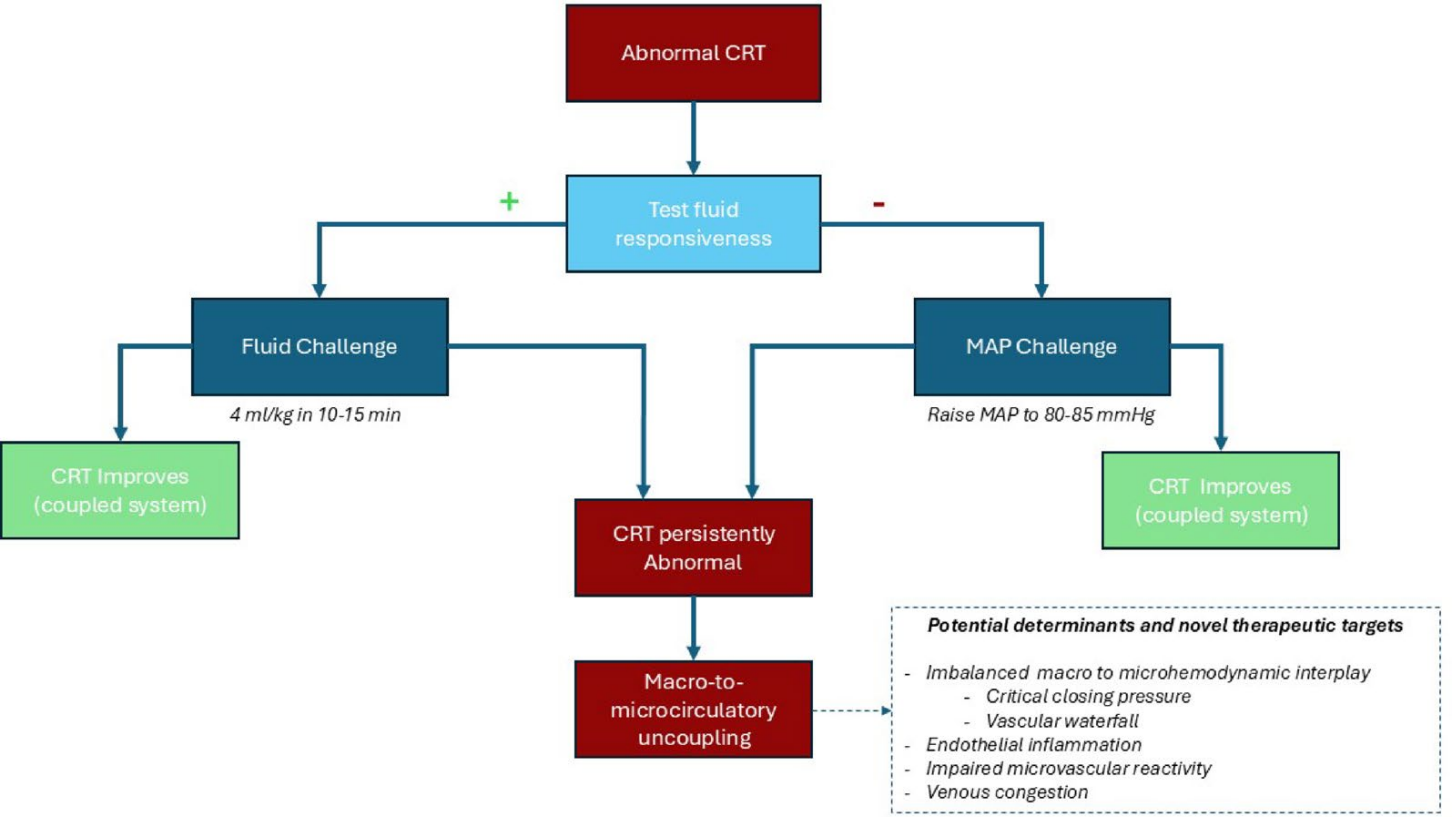
Simple hypothetical algorithm to test the status of macro-to microcirculatory coupling according to capillary refill time response to common resuscitative interventions. *CRT* capillary refill time, *MAP* mean arterial pressure

The ANDROMEDA-SHOCK trial introduced the concept of a MAP challenge (or vasopressor test) to assess the integrity of macro-to-microcirculatory coupling [9]. A positive CRT response to increased MAP may help guide clinicians in favoring a higher MAP target. More importantly, identifying patients whose CRT worsens with increased vasopressor use can be critical for avoiding excessive vasoconstriction and may support adopting lower MAP goals. If validated by further research, this bedside MAP challenge could become a valuable tool for personalizing hemodynamic targets in septic shock. Nonetheless, understanding the determinants of individual responses remains limited. The administration of higher norepinephrine doses may influence both MAP and CO, and their impact on lesser-known physiological factors—such as critical closing pressure or the vascular waterfall effect—warrant further investigation. Figure 3 depicts a potential way to understand the intricate relationship between these concepts.

### CRT role during de-escalation

CRT has also shown promise as a tool to guide fluid management during renal replacement therapy. In a pilot study involving patients receiving continuous renal replacement therapy, a de-resuscitation strategy guided by CRT and mottling score was associated with more negative fluid balances without an increase in hemodynamic instability [63]. Similarly, in the SOCRATE study, a CRT > 3 s prior to the initiation of intermittent hemodialysis was independently associated with a higher risk of intradialytic hemodynamic instability [64].

### CRT limitations and research agenda

CRT has traditionally been considered a subjective assessment of peripheral perfusion [65], and as such, many issues regarding its validity has been raised. A wide range of patient-specific factors, such as age, sex, core temperature [66–68], as well as environmental factors, such as ambient temperature and lighting [66, 69] can affect this clinical parameter. Moreover, practices regarding its implementation vary across settings and studies, including differences in anatomical sites of measurement [70–73], pressure application strength and duration [67, 74], among others. Even when controlling for these variables or measuring within the same patient, operator-specific factors may hinder its internal validity for research, with significant intra-observer and inter-observer variability reported [75–78]. Potential sources have been studied, such as the training level of the operator [79].

In response to these limitations, efforts have been made to standardize its application, with differents methods proposed for its correct measurement [17, 26, 27, 71]. These methods improved intra- and inter-observer reliability, measured by higher intraclass correlation coefficients. A recently published technical note by Hernandez et al. proposed a standardized and clinically aplicable method for both daily practice and research endeavours [11], supported by data from a large randomized control trial [9]. To further address these limitations in research applications, which require a low variability to ensure high internal validity, semi-automated and fully automated CRT measurement devices have been developed [80–85]. Most of these rely on either photo-plethysmogramy or video evaluation, requiring manual or pneumatic compression, and have shown greater reliability [86, 87]. Nevertheless, the translation of the demostrated benefits of manual CRT monitoring to these devices should be validated in future prospective studies.

Within the limitations of this parameter is that the kinetics and determinants of CRT have not been adequately investigated in patients with peripheral vasculopathies or cirrhosis, both of which involve distinct alterations in vascular physiology. In peripheral vasculopathies, local blood flow may be impaired due to structural vascular abnormalities [88–90], potentially affecting CRT independently of systemic hemodynamic status. Similarly, in cirrhosis, profound changes in vascular tone, splanchnic vasodilation, and circulatory dysregulation may alter peripheral perfusion dynamics [91, 92]. As a result, the relationship between CRT and systemic hemodynamics described in this review may not be directly applicable to these patient populations. Further studies are needed to characterize CRT behavior and its relationship with macrohemodynamics in these specific clinical contexts. Finally, the role of venous congestion as a driver of abnormal tissue perfusion should be further explored [47, 93].

## Conclusions

From a physiological point of view, CRT is a complex variable integrating microvascular flow and reactivity. It should also be understood as a dynamic test that evaluates the preservation or disruption of normal responses of the microcirculation to maintain blood flow after transient ischemic challenges.

The relationship between systemic hemodynamics and CRT is complex. Indeed, single time-point asssessments of CRT are not able to predict absolute cardiac output values and this is logical since they belong to different phsyiological categories. An abnormal CRT may be explained by insufficient macrohemodynamic resuscitation but also by several derangements at the microvascular level that may preclude reperfusion and thus CRT normalization, signaling a state of macro-to-microcirculatory uncoupling.

CRT response to an acute fluid or mean arterial pressure challenge, may not only reveal the adequacy of systemic blood flow but also contribute to tailor interventions to personalize septic shock resuscitation. The lack of CRT response to these challenges discloses a more complex pathophysiological condition that is associated with higher mortality. Further research efforts should be focused on better understanding the factors associated with CRT non-response as a first step to develop a more phsyiologically-based resuscitation, that could eventually improve outcomes.

## Data Availability

Not applicable.

## Abbreviations

CRT: Capillary refill time
CO: Cardiac output
MAP: Mean arterial pressure
CI: Cardiac index

## References

[1] Bakker J, Kattan E, Annane D, Castro R, Cecconi M, De Backer D, et al. Current practice and evolving concepts in septic shock resuscitation. Intensive Care Med. 2022;48:148–63. 34910228 10.1007/s00134-021-06595-9

[2] Kattan E, Castro R, Vera M, Hernández G. Optimal target in septic shock resuscitation. Ann Transl Med. 2020;8:789–789. 32647714 10.21037/atm-20-1120 PMC7333135

[3] Meyer NJ, Prescott HC. Sepsis and Septic Shock. Hardin CC, editor. N Engl J Med. 2024;391:2133–46. 10.1056/NEJMra2403213 39774315

[4] De Backer D, Cecconi M, Chew MS, Hajjar L, Monnet X, Ospina-Tascón GA, et al. A plea for personalization of the hemodynamic management of septic shock. Crit Care. 2022;26:372. 36457089 10.1186/s13054-022-04255-y PMC9714237

[5] Hernandez G, Luengo C, Bruhn A, Kattan E, Friedman G, Ospina-Tascon GA, et al. When to stop septic shock resuscitation: clues from a dynamic perfusion monitoring. Ann Intensive Care. 2014;4:30. 25593746 10.1186/s13613-014-0030-z PMC4273696

[6] Hernandez G, Bruhn A, Castro R, Regueira T. The holistic view on perfusion monitoring in septic shock. Curr Opin Crit Care. 2012;18:280–6. 22473257 10.1097/MCC.0b013e3283532c08

[7] Hernández G, Teboul J-L. Is the macrocirculation really dissociated from the microcirculation in septic shock? Intensive Care Med. 2016;42:1621–4. 27289357 10.1007/s00134-016-4416-2

[8] Guedel AE. Cyclopropane Anesth Anesthesiology. 1940;1:13–25.

[9] Hernández G, Ospina-Tascón GA, Damiani LP, Estenssoro E, Dubin A, Hurtado J, et al. Effect of a resuscitation strategy targeting peripheral perfusion status vs serum lactate levels on 28-Day mortality among patients with septic shock: the ANDROMEDA-SHOCK randomized clinical trial. JAMA. 2019;321:654. 30772908 10.1001/jama.2019.0071 PMC6439620

[10] Bloch JH, Dietzman RH, Pierce CH, Lillehei RC. Theories of the production of shock. Br J Anaesth. 1966;38:234–49. 5328405 10.1093/bja/38.4.234

[11] Hernandez G, Carmona P, Ait-Oufella H. Monitoring capillary refill time in septic shock. Intensive Care Med. 2024;50:580–2. 38498167 10.1007/s00134-024-07361-3

[12] Kattan E, Ibarra-Estrada M, Ospina-Tascón G, Hernández G. Perspectives on peripheral perfusion assessment. Curr Opin Crit Care. 2023;29:208–14. 37078639 10.1097/MCC.0000000000001038

[13] Hariri G, Joffre J, Leblanc G, Bonsey M, Lavillegrand J-R, Urbina T, et al. Narrative review: clinical assessment of peripheral tissue perfusion in septic shock. Ann Intensive Care. 2019;9:37. 30868286 10.1186/s13613-019-0511-1 PMC6419794

[14] Wang M, Tong M, Tian Z. Prolonged capillary refill time and short-term mortality of critically ill patients: A meta-analysis. Am J Emerg Med. 2024;79:127–35. 38430706 10.1016/j.ajem.2024.01.041

[15] Jacquet-Lagrèze M, Pernollet A, Kattan E, Ait-Oufella H, Chesnel D, Ruste M, et al. Prognostic value of capillary refill time in adult patients: a systematic review with meta-analysis. Crit Care. 2023;27:473. 38042855 10.1186/s13054-023-04751-9 PMC10693708

[16] Contreras R, Hernández G, Valenzuela ED, González C, Ulloa R, Soto D, et al. Exploring the relationship between capillary refill time, skin blood flow and microcirculatory reactivity during early resuscitation of patients with septic shock: a pilot study. J Clin Monit Comput. 2023;37:839–45. 36495360 10.1007/s10877-022-00946-7

[17] Morin A, Missri L, Urbina T, Bonny V, Gasperment M, Bernier J, et al. Relationship between skin microvascular blood flow and capillary refill time in critically ill patients. Crit Care. 2025;29:57. 39905546 10.1186/s13054-025-05285-y PMC11792347

[18] SICS Study Group, Hiemstra B, Koster G, Wiersema R, Hummel YM, Van Der Harst P, et al. The diagnostic accuracy of clinical examination for estimating cardiac index in critically ill patients: the simple intensive care Studies-I. Intensive Care Med. 2019;45:190–200. 30706120 10.1007/s00134-019-05527-y

[19] Hernández G, Valenzuela ED, Kattan E, Castro R, Guzmán C, Kraemer AE, et al. Capillary refill time response to a fluid challenge or a vasopressor test: an observational, proof-of-concept study. Ann Intensive Care. 2024;14:49. 38558268 10.1186/s13613-024-01275-5 PMC10984906

[20] Kirchheim HR, Ehmke H, Hackenthal E, Löwe W, Persson P. Autoregulation of renal blood flow, glomerular filtration rate and Renin release in conscious dogs. Pflüg Arch - Eur J Physiol. 1987;410:441–9. 10.1007/BF00586523 3324052

[21] Stainsby WN. Autoregulation of blood flow in skeletal muscle during increased metabolic activity. Am J Physiol-Leg Content. 1962;202:273–6. 10.1152/ajplegacy.1962.202.2.273 13916114

[22] Norris CP, Barnes GE, Smith EE, Granger HJ. Autoregulation of superior mesenteric flow in fasted and fed dogs. Am J Physiol-Heart Circ Physiol. 1979;237:H174–7. 10.1152/ajpheart.1979.237.2.H174 464109

[23] Doerschug KC, Delsing AS, Schmidt GA, Haynes WG. Impairments in microvascular reactivity are related to organ failure in human sepsis. Am J Physiol-Heart Circ Physiol. 2007;293:H1065–71. 17483235 10.1152/ajpheart.01237.2006

[24] Neviere R, Mathieu D, Chagnon JL, Lebleu N, Millien JP, Wattel F. Skeletal muscle microvascular blood flow and oxygen transport in patients with severe sepsis. Am J Respir Crit Care Med. 1996;153:191–5. 8542115 10.1164/ajrccm.153.1.8542115

[25] Brunauer A, Koköfer A, Bataar O, Gradwohl-Matis I, Dankl D, Bakker J, et al. Changes in peripheral perfusion relate to visceral organ perfusion in early septic shock: A pilot study. J Crit Care. 2016;35:105–9. 27481743 10.1016/j.jcrc.2016.05.007

[26] Jacquet-Lagrèze M, Bouhamri N, Portran P, Schweizer R, Baudin F, Lilot M, et al. Capillary refill time variation induced by passive leg Raising predicts capillary refill time response to volume expansion. Crit Care. 2019;23:281. 31420052 10.1186/s13054-019-2560-0 PMC6697974

[27] Raia L, Gabarre P, Bonny V, Urbina T, Missri L, Boelle P-Y, et al. Kinetics of capillary refill time after fluid challenge. Ann Intensive Care. 2022;12:74. 35962860 10.1186/s13613-022-01049-x PMC9375797

[28] Fage N, Moretto F, Rosalba D, Shi R, Lai C, Teboul J-L, et al. Effect on capillary refill time of volume expansion and increase of the norepinephrine dose in patients with septic shock. Crit Care. 2023;27:429. 37932812 10.1186/s13054-023-04714-0 PMC10629142

[29] Maitland K, Pamba A, Newton CRJC, Levin M. Response to volume resuscitation in children with severe malaria*. Pediatr Crit Care Med. 2003;4:426–31. 14525636 10.1097/01.PCC.0000090293.32810.4E

[30] McGee S. Is this Patient Hypovolemic? JAMA. 1999;281:1022. 10086438 10.1001/jama.281.11.1022

[31] Schriger DL, Baraff LJ. Capillary refill — is it a useful predictor of hypovolemic states? Ann Emerg Med. 1991;20:601–5. 2039096 10.1016/s0196-0644(05)82375-3

[32] Gross CR, Lindquist RD, Woolley AC, Granieri R, Allard K, Webster B. Clinical indicators of dehydration severity in elderly patients. J Emerg Med. 1992;10:267–74. 1624737 10.1016/0736-4679(92)90331-m

[33] Gustavsson H, Meyer F, Fahlander S, Ölwegård B, Jonasson H, Toll R, et al. Capillary refill time paradoxically decreases in a blood loss shock model. Intensive Care Med Exp. 2025;13:8. 39841394 10.1186/s40635-025-00714-2 PMC11754564

[34] The ANDROMEDA-SHOCK Study Investigators and the Latin America Intensive Care Network (LIVEN), Hernández G, Kattan E, Ospina-Tascón G, Bakker J, Castro R. Capillary refill time status could identify different clinical phenotypes among septic shock patients fulfilling Sepsis-3 criteria: a post hoc analysis of ANDROMEDA-SHOCK trial. Intensive Care Med. 2020;46:816–8. 32076766 10.1007/s00134-020-05960-4

[35] Kattan E, Hernández G, Ospina-Tascón G, Valenzuela ED, Bakker J, Castro R, et al. A lactate-targeted resuscitation strategy May be associated with higher mortality in patients with septic shock and normal capillary refill time: a post hoc analysis of the ANDROMEDA-SHOCK study. Ann Intensive Care. 2020;10:114. 32845407 10.1186/s13613-020-00732-1 PMC7450018

[36] Hernández G, Castro R, Bakker J. Capillary refill time: the missing link between macrocirculation and microcirculation in septic shock? J Thorac Dis. 2020;12:1127–9. 32274184 10.21037/jtd.2019.12.102 PMC7139032

[37] Ince C. Hemodynamic coherence and the rationale for monitoring the microcirculation. Crit Care. 2015;19:S8. 26729241 10.1186/cc14726 PMC4699073

[38] De Backer D, Creteur J, Preiser J-C, Dubois M-J, Vincent J-L. Microvascular blood flow is altered in patients with sepsis. Am J Respir Crit Care Med. 2002;166:98–104. 12091178 10.1164/rccm.200109-016oc

[39] Ince C. The microcirculation is the motor of sepsis. Crit Care. 2005;9:S13. 16168069 10.1186/cc3753 PMC3226164

[40] Bruno RR, Hernandez G, Thiele H, Kattan E, Jung C. The DAMIS study group. A microcirculation-guided trial: never trying is worse than failing. Intensive Care Med. 2023;49:1555–6. 37812227 10.1007/s00134-023-07245-y PMC10709223

[41] Bruno RR, Schemmelmann M, Hornemann J, Moecke HME, Demirtas F, Palici L, et al. Sublingual microcirculatory assessment on admission independently predicts the outcome of old intensive care patients suffering from shock. Sci Rep. 2024;14:25668. 39463395 10.1038/s41598-024-77357-y PMC11514226

[42] Ospina-Tascon G, Neves AP, Occhipinti G, Donadello K, Büchele G, Simion D, et al. Effects of fluids on microvascular perfusion in patients with severe sepsis. Intensive Care Med. 2010;36:949–55. 20221744 10.1007/s00134-010-1843-3

[43] On behalf of the Cardiovascular Dynamics Section of the ESICM, Ince C, Boerma EC, Cecconi M, De Backer D, Shapiro NI, et al. Second consensus on the assessment of Sublingual microcirculation in critically ill patients: results from a task force of the European society of intensive care medicine. Intensive Care Med. 2018;44:281–99. 29411044 10.1007/s00134-018-5070-7

[44] Permutt S, Riley RL. Hemodynamics of collapsible vessels with tone: the vascular waterfall. J Appl Physiol. 1963;18:924–32. 14063262 10.1152/jappl.1963.18.5.924

[45] Pinsky MR, García MIM, Dubin A. Significance of critical closing pressures (starling resistors) in arterial circulation. Crit Care. 2024;28:127. 38637877 10.1186/s13054-024-04912-4 PMC11025166

[46] Sanchez EC, Taha A, Tolba Y, Hernandez G, Pinsky MR. Assessment of Tissue Perfusion Pressure in Patients With Septic Shock: Beyond Mean Arterial Pressure. Crit Care Med [Internet]. 2025 [cited 2025 July 31]; Available from: https://journals.lww.com/10.1097/CCM.0000000000006805 10.1097/CCM.0000000000006805 40668153

[47] Rola P, Kattan E, Siuba MT, Haycock K, Crager S, Spiegel R, et al. Point of view: A holistic Four-Interface conceptual model for personalizing shock resuscitation. J Pers Med. 2025;15:207. 40423078 10.3390/jpm15050207 PMC12113614

[48] Hayes MA, Timmins AC, Yau E, Palazzo M, Hinds CJ, Watson D. Elevation of systemic oxygen delivery in the treatment of critically ill patients. N Engl J Med. 1994;330:1717–22. 7993413 10.1056/NEJM199406163302404

[49] Castro R, Kattan E, Ferri G, Pairumani R, Valenzuela ED, Alegría L, et al. Effects of capillary refill time-vs. lactate-targeted fluid resuscitation on regional, microcirculatory and hypoxia-related perfusion parameters in septic shock: a randomized controlled trial. Ann Intensive Care. 2020;10:150. 33140173 10.1186/s13613-020-00767-4 PMC7606372

[50] Bennett VA, Vidouris A, Cecconi M. Effects of fluids on the Macro- and microcirculations. Crit Care. 2018;22:74. 29558989 10.1186/s13054-018-1993-1 PMC5861604

[51] Gabarre P, Desnos C, Morin A, Missri L, Urbina T, Bonny V, et al. Albumin versus saline infusion for sepsis-related peripheral tissue hypoperfusion: a proof-of-concept prospective study. Crit Care. 2024;28:43. 38326920 10.1186/s13054-024-04827-0 PMC10848485

[52] Cusack R, O’Neill S, Martin-Loeches I. Effects of fluids on the Sublingual microcirculation in sepsis. J Clin Med. 2022;11:7277. 36555895 10.3390/jcm11247277 PMC9786137

[53] Evans L, Rhodes A, Alhazzani W, Antonelli M, Coopersmith CM, French C, et al. Surviving sepsis campaign: international guidelines for management of sepsis and septic shock 2021. Intensive Care Med. 2021;47:1181–247. 34599691 10.1007/s00134-021-06506-y PMC8486643

[54] Lamontagne F, Day AG, Meade MO, Cook DJ, Guyatt GH, Hylands M, et al. Pooled analysis of higher versus lower blood pressure targets for vasopressor therapy septic and vasodilatory shock. Intensive Care Med. 2018;44:12–21. 29260272 10.1007/s00134-017-5016-5

[55] Lamontagne F, Richards-Belle A, Thomas K, Harrison DA, Sadique MZ, Grieve RD, et al. Effect of reduced exposure to vasopressors on 90-Day mortality in older critically ill patients with vasodilatory hypotension: A randomized clinical trial. JAMA. 2020;323:938. 32049269 10.1001/jama.2020.0930 PMC7064880

[56] Asfar P, Meziani F, Hamel J-F, Grelon F, Megarbane B, Anguel N, et al. High versus low Blood-Pressure target in patients with septic shock. N Engl J Med. 2014;370:1583–93. 24635770 10.1056/NEJMoa1312173

[57] Dünser MW, Takala J, Brunauer A, Bakker J. Re-thinking resuscitation: leaving blood pressure cosmetics behind and moving forward to permissive hypotension and a tissue perfusion-based approach. Crit Care. 2013;17:326. 24103466 10.1186/cc12727 PMC4056569

[58] Endo A, Yamakawa K, Tagami T, Umemura Y, Wada T, Yamamoto R, et al. Efficacy of targeting high mean arterial pressure for older patients with septic shock (OPTPRESS): a multicentre, pragmatic, open-label, randomised controlled trial. Intensive Care Med. 2025;51:883–92. 40358717 10.1007/s00134-025-07910-4 PMC12130109

[59] Angriman F, Momenzade N, Adhikari NKJ, Mouncey PR, Asfar P, Yarnell CJ, et al. Blood pressure targets for adults with vasodilatory Shock — An individual patient data Meta-Analysis. NEJM Evid. 2025;4:1. 10.1056/EVIDoa2400359 PMC12289332 39556565

[60] Thooft A, Favory R, Salgado DR, Taccone FS, Donadello K, De Backer D, et al. Effects of changes in arterial pressure on organ perfusion during septic shock. Crit Care. 2011;15:R222. 21936903 10.1186/cc10462 PMC3334768

[61] Dubin A, Pozo MO, Casabella CA, Pálizas F, Murias G, Moseinco MC, et al. Increasing arterial blood pressure with norepinephrine does not improve microcirculatory blood flow: a prospective study. Crit Care. 2009;13:R92. 19534818 10.1186/cc7922 PMC2717464

[62] Andrei S, Bar S, Nguyen M, Bouhemad B, Guinot P-G. Effect of norepinephrine on the vascular waterfall and tissue perfusion in vasoplegic hypotensive patients: a prospective, observational, applied physiology study in cardiac surgery. Intensive Care Med Exp. 2023;11:52. 37599310 10.1186/s40635-023-00539-x PMC10440321

[63] Ruste M, Sghaier R, Chesnel D, Didier L, Fellahi J-L, Jacquet-Lagrèze M. Perfusion-based deresuscitation during continuous renal replacement therapy: A before-after pilot study (The early dry Cohort). J Crit Care. 2022;72:154169. 36201978 10.1016/j.jcrc.2022.154169

[64] Bigé N, Lavillegrand J-R, Dang J, Attias P, Deryckere S, Joffre J, et al. Bedside prediction of intradialytic hemodynamic instability in critically ill patients: the SOCRATE study. Ann Intensive Care. 2020;10:47. 32323060 10.1186/s13613-020-00663-x PMC7176798

[65] Lima A, Jansen TC, Van Bommel J, Ince C, Bakker J. The prognostic value of the subjective assessment of peripheral perfusion in critically ill patients. Crit Care Med. 2009;37:934–8. 19237899 10.1097/CCM.0b013e31819869db

[66] Anderson B, Kelly A-M, Kerr D, Clooney M, Jolley D. Impact of patient and environmental factors on capillary refill time in adults. Am J Emerg Med. 2008;26:62–5. 18082783 10.1016/j.ajem.2007.06.026

[67] Pickard A, Karlen W, Ansermino JM. Capillary refill time: is it still a useful clinical sign?? Anesth Analg. 2011;113:120–3. 21519051 10.1213/ANE.0b013e31821569f9

[68] Schriger DL, Baraff L. Defining normal capillary refill: variation with age, sex, and temperature. Ann Emerg Med. 1988;17:932–5. 3415066 10.1016/s0196-0644(88)80675-9

[69] Brown LH, Prasad NH, Whitley TW. Adverse lighting condition effects on the assessment of capillary refill. Am J Emerg Med. 1994;12:46–7. 8285971 10.1016/0735-6757(94)90196-1

[70] Jacquet-Lagrèze M, Wiart C, Schweizer R, Didier L, Ruste M, Coutrot M, et al. Capillary refill time for the management of acute circulatory failure: a survey among pediatric and adult intensivists. BMC Emerg Med. 2022;22:131. 35850662 10.1186/s12873-022-00681-x PMC9290243

[71] Ait-Oufella H, Bige N, Boelle PY, Pichereau C, Alves M, Bertinchamp R, et al. Capillary refill time exploration during septic shock. Intensive Care Med. 2014;40:958–64. 24811942 10.1007/s00134-014-3326-4

[72] Crook J, Taylor RM. The agreement of fingertip and sternum capillary refill time in children. Arch Dis Child. 2013;98:265–8. 23396679 10.1136/archdischild-2012-303046

[73] Lobos A-T, Menon K. A multidisciplinary survey on capillary refill time: inconsistent performance and interpretation of a common clinical test. Pediatr Crit Care Med. 2008;9:386–91. 18496415 10.1097/PCC.0b013e3181728798

[74] Kawaguchi R, Nakada T, Oshima T, Shinozaki M, Nakaguchi T, Haneishi H, et al. Optimal pressing strength and time for capillary refilling time. Crit Care. 2019;23:4. 30621748 10.1186/s13054-018-2295-3 PMC6323707

[75] Espinoza EDV, Welsh S, Dubin A. Lack of agreement between different observers and methods in the measurement of capillary refill time in healthy volunteers: an observational study. Rev Bras Ter Intensiva. 2014;26:269–76. 25295821 10.5935/0103-507X.20140038 PMC4188463

[76] Anderson B, Kelly A, Kerr D, Jolley D. Capillary refill time in adults has poor Inter-Observer agreement. Hong Kong J Emerg Med. 2008;15:71–4.

[77] Brabrand M, Hosbond S, Folkestad L. Capillary refill time: a study of interobserver reliability among nurses and nurse assistants. Eur J Emerg Med. 2011;18:46–9. 20512037 10.1097/MEJ.0b013e32833b4fba

[78] Alsma J, Van Saase JLCM, Nanayakkara PWB, Schouten WEMI, Baten A, Bauer MP, et al. The power of flash mob research. Chest. 2017;151:1106–13. 27940191 10.1016/j.chest.2016.11.035

[79] Shinozaki K, Jacobson LS, Saeki K, Kobayashi N, Weisner S, Falotico JM, et al. Does training level affect the accuracy of visual assessment of capillary refill time? Crit Care. 2019;23:157. 31060576 10.1186/s13054-019-2444-3 PMC6501297

[80] Kviesis-Kipge E, Curkste E, Spigulis J, Gardovska D. Optical Studies of the Capillary Refill Kinetics in Fingertips. In: Dössel O, Schlegel WC, editors. World Congr Med Phys Biomed Eng Sept 7–12 2009 Munich Ger [Internet]. Berlin, Heidelberg: Springer Berlin Heidelberg; 2009 [cited 2025 July 31]. pp. 377–9. Available from: http://link.springer.com/10.1007/978-3-642-03885-3_105

[81] Shavit I, Brant R, Nijssen-Jordan C, Galbraith R, Johnson DW. A novel imaging technique to measure Capillary-Refill time: improving diagnostic accuracy for dehydration in young children with gastroenteritis. Pediatrics. 2006;118:2402–8. 17142525 10.1542/peds.2006-1108

[82] Blaxter LL, Morris DE, Crowe JA, Henry C, Hill S, Sharkey D, et al. An automated quasi-continuous capillary refill timing device. Physiol Meas. 2016;37:83–99. 26642080 10.1088/0967-3334/37/1/83 PMC4770525

[83] Shinozaki K, Jacobson LS, Saeki K, Hirahara H, Kobayashi N, Weisner S, et al. Comparison of point-of-care peripheral perfusion assessment using pulse oximetry sensor with manual capillary refill time: clinical pilot study in the emergency department. J Intensive Care. 2019;7:52. 31798887 10.1186/s40560-019-0406-0 PMC6880499

[84] Jacquet-Lagrèze M, Saint-Jean C, Bouët T, Reynaud S, Ruste M, Fellahi J-L. Reliability and reproducibility of the DICART device to assess capillary refill time: a bench and in-silico study. J Clin Monit Comput. 2023;37:1409–12. 37199880 10.1007/s10877-023-01027-z

[85] Shinozaki M, Saito D, Nakada T, Nomura Y, Nakaguchi T. Feasibility study of wearable capillary refill time measurement device. Artif Life Robot. 2024;29:334–9.

[86] Shinozaki K, Saeki K, Jacobson LS, Falotico JM, Li T, Hirahara H, et al. Evaluation of accuracy of capillary refill index with pneumatic fingertip compression. J Clin Monit Comput. 2021;35:135–45. 31916222 10.1007/s10877-019-00454-1

[87] Descamps A, Jacquet-Lagrèze M, Aussal T, Fellahi J-L, Ruste M. DiCARTTM device to measure capillary refill time: a validation study in patients with acute circulatory failure. J Clin Monit Comput [Internet]. 2025 [cited 2025 July 31]; Available from: https://link.springer.com/10.1007/s10877-025-01271-5 10.1007/s10877-025-01271-5 PMC12474624 40011397

[88] Meneses AL, Nam MCY, Bailey TG, Magee R, Golledge J, Hellsten Y, et al. Leg blood flow and skeletal muscle microvascular perfusion responses to submaximal exercise in peripheral arterial disease. Am J Physiol-Heart Circ Physiol. 2018;315:H1425–33. 30095999 10.1152/ajpheart.00232.2018

[89] Wu W-C, Mohler E, Ratcliffe SJ, Wehrli FW, Detre JA, Floyd TF. Skeletal muscle microvascular flow in progressive peripheral artery disease. J Am Coll Cardiol. 2009;53:2372–7. 19539149 10.1016/j.jacc.2009.03.033 PMC2763280

[90] Bethel M, Annex BH. Peripheral arterial disease: A small and large vessel problem. Am Heart J Plus Cardiol Res Pract. 2023;28:100291. 10.1016/j.ahjo.2023.100291 PMC10945902 38511071

[91] La Villa G, Gentilini P. Hemodynamic alterations in liver cirrhosis. Mol Aspects Med. 2008;29:112–8. 18177931 10.1016/j.mam.2007.09.010

[92] Hori T, Ogura Y, Onishi Y, Kamei H, Kurata N, Kainuma M, et al. Systemic hemodynamics in advanced cirrhosis: concerns during perioperative period of liver transplantation. World J Hepatol. 2016;8:1047. 27660671 10.4254/wjh.v8.i25.1047 PMC5026996

[93] Ruste M, Reskot R, Schweizer R, Mayet V, Fellahi J-L, Jacquet-Lagrèze M. Changes in portal pulsatility index induced by a fluid challenge in patients with haemodynamic instability and systemic venous congestion: a prospective cohort study. Ann Intensive Care. 2024;14:167. 39485575 10.1186/s13613-024-01391-2 PMC11530414

